# Frog Oocytes to Unveil the Structure and Supramolecular Organization of Human Transport Proteins

**DOI:** 10.1371/journal.pone.0021901

**Published:** 2011-07-07

**Authors:** Marc J. Bergeron, Rajendra Boggavarapu, Marcel Meury, Zöhre Ucurum, Luc Caron, Paul Isenring, Matthias A. Hediger, Dimitrios Fotiadis

**Affiliations:** 1 Institute of Biochemistry and Molecular Medicine, and Swiss National Centre of Competence in Research (NCCR) TransCure, University of Bern, Bern, Switzerland; 2 Unité de Neurobiologie Cellulaire, Centre de Recherche Université Laval Robert-Giffard, Québec, Canada; 3 Nephrology Research Group, CHUQ-L'Hôtel-Dieu de Québec Institution, Department of Medicine, Faculty of Medicine, Laval University, Québec, Canada; Swiss Federal Institute of Technology Zurich, Switzerland

## Abstract

Structural analyses of heterologously expressed mammalian membrane proteins remain a great challenge given that microgram to milligram amounts of correctly folded and highly purified proteins are required. Here, we present a novel method for the expression and affinity purification of recombinant mammalian and in particular human transport proteins in *Xenopus laevis* frog oocytes. The method was validated for four human and one murine transporter. Negative stain transmission electron microscopy (TEM) and single particle analysis (SPA) of two of these transporters, i.e., the potassium-chloride cotransporter 4 (KCC4) and the aquaporin-1 (AQP1) water channel, revealed the expected quaternary structures within homogeneous preparations, and thus correct protein folding and assembly. This is the first time a cation-chloride cotransporter (SLC12) family member is isolated, and its shape, dimensions, low-resolution structure and oligomeric state determined by TEM, i.e., by a direct method. Finally, we were able to grow 2D crystals of human AQP1. The ability of AQP1 to crystallize was a strong indicator for the structural integrity of the purified recombinant protein. This approach will open the way for the structure determination of many human membrane transporters taking full advantage of the *Xenopus laevis* oocyte expression system that generally yields robust functional expression.

## Introduction

The number of membrane proteins for which high-resolution 3D structures have been published is still low, amounting to 293 unique structures as of June 2011 (http://blanco.biomol.uci.edu/Membrane_Proteins_xtal.html) in contrast to over ten thousand structures of water-soluble proteins. Among these unique structures, most are of bacterial membrane proteins expressed in bacteria or of eukaryotic membrane proteins expressed at unusually high levels in their natural environment. Otherwise, only ∼20 eukaryotic structures are of recombinant membrane proteins [1].

The major bottleneck for structural studies of mammalian membrane proteins is the production of micrograms to milligrams of highly purified and correctly folded protein implying that heterologous overexpression will very often be mandatory. For high-resolution structure determination by electron and X-ray crystallography, milligram amounts of protein are required to grow well-diffracting 2D and 3D protein crystals. On the other hand, for low- (10–30 Å) and medium-resolution (<10 Å) structure determination by negative stain and TEM of single particles or of moderately ordered 2D crystals, microgram amounts of protein are generally sufficient. Such low- and medium-resolution structures are nonetheless informative given that they reveal the arrangement of transmembrane alpha-helices and other secondary structure elements within membrane proteins as well as their supramolecular assembly.

Expression systems currently used to produce membrane proteins for structural studies include bacteria, yeast, insect cells, cell-free approaches and mammalian cells. Each system has its advantages but none is optimal for all types of membrane proteins. The *Xenopus laevis* oocyte expression system could represent one exception given that it has been shown to allow for the robust expression of many functional mammalian channels and solute carriers (SLCs) [2]. This system owes its success to its ability to translate heterologous mRNA and cDNA-derived cRNA efficiently, and to provide most of the necessary cofactors required for the functional expression of recombinant proteins at the cell surface. Due to technical and methodological limitations, however, there have been no reports describing the purification of recombinant mammalian membrane proteins from oocytes, e.g. for structural studies.

In the current study, *X. laevis* oocytes were used to express recombinant mammalian (in particular human) transport proteins for their subsequent purification and structural characterization. Channels and SLCs were taken as model proteins because they represent the majority of transport proteins, are linked to numerous inherited and acquired human diseases and correspond to key therapeutic targets. Purification was achieved by expressing recombinant proteins tagged with multiple epitopes and by using a novel procedure for the preparation of egg yolk-depleted total membranes. These two features were crucial for the successful purification of transport proteins.

Five transport systems were purified in microgram amounts using the novel method: aquaporin-1 (AQP1), glutamate transporter 1 (EAAC1 or SLC1A1), peptide transporter 1 (PEPT1 or SLC15A1) and sodium-glucose-cotransporter 1 (SGLT1 or SLC5A1) from human, and potassium-chloride cotransporter 4 (KCC4 or SLC12A7) from mouse. To validate our approach, we first tested the expression, localization and function of recombinant AQP1 and KCC4 in oocytes. Negative stain TEM and SPA of purified AQP1 and KCC4 indicated homogenous particle distributions and the expected oligomeric states. From the purification procedure described here, lastly, it was possible to grow 2D crystals of human AQP1 expressed in *Xenopus laevis* oocytes, paving the way for future structural analyses of mammalian membrane proteins by crystallography techniques.

## Results

### Design of the expression vector and workflow

**Figure 1:**
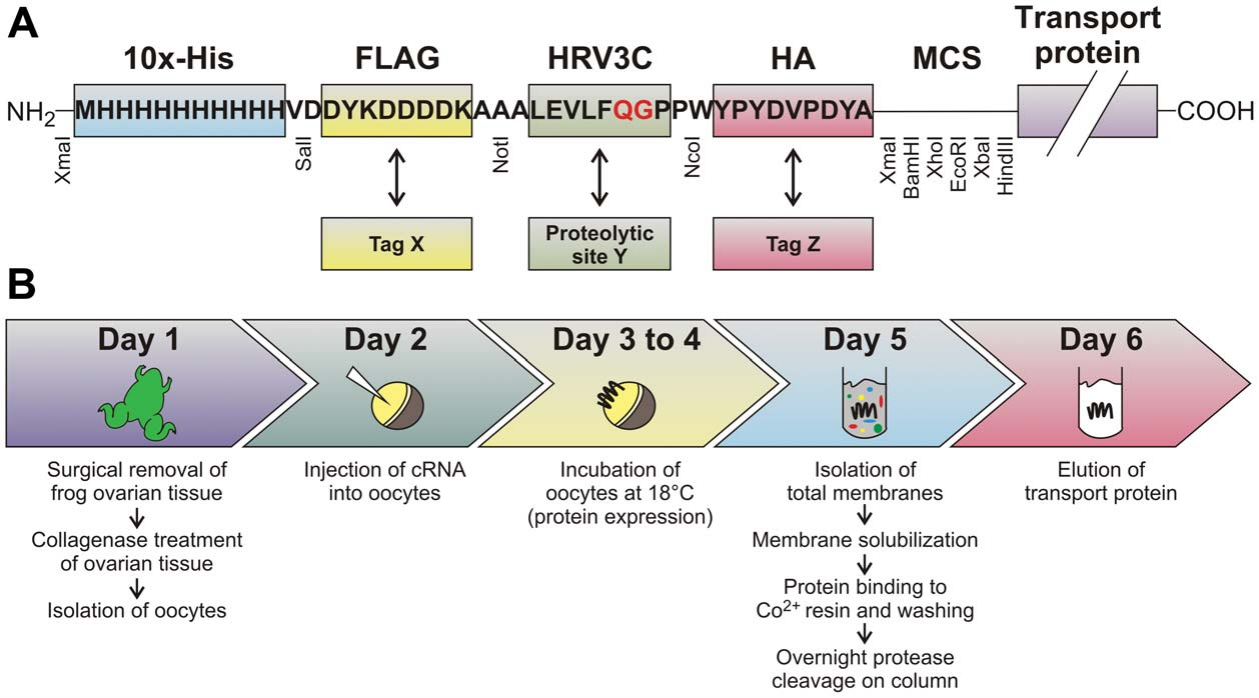
Schematic representation of the expression vector pMJB08, and the workflow for the expression in *X. laevis* oocytes and purification of mammalian transport proteins. (A) Three tags, i.e. 10x-His, FLAG and HA, and one proteolytic site for the HRV3C protease were translated in-frame at the N-terminus of our transport proteins. The HRV3C protease cleaves between the amino acids QG (in red). The vector is setup in a modular manner: *i.)* the FLAG tag (yellow) can be swapped by a different tag X (e.g. *c-myc*, HA, etc.) using the unique restriction sites SalI and NotI; *ii.)* the HRV3C protease cleavage site (green) can be replaced by a different proteolytic site Y (e.g. for TEV protease, thrombin, etc.) using the unique restriction sites NotI and NcoI; *iii.)* the HA tag (red) can be exchanged by a different tag Z (e.g. *c-myc*, FLAG, etc.) using the unique restriction sites NcoI and all enzymes in the multi cloning site (MCS) except XmaI; *iv.)* a XmaI digestion removes all tags and epitopes to obtain the original Pol1 vector. (B) Schematic representation of the workflow for the expression and purification of transport proteins in *X. laevis* oocytes. See Methods section for a detailed description of the different steps.

The oocyte expression vector Pol1 [3] was modified by adding the decahistidine (10x-His), FLAG and hemagglutinin (HA) epitope tags in front of the multiple cloning site (Figure 1A). A cleavage site for the human rhinovirus 3C (HRV3C) protease (also known as PreScission™) was inserted between the FLAG and HA tags to remove the His and FLAG tags from purified proteins. Importantly, each of the epitope and proteolytic cleavage modules was flanked by convenient restriction sites to allow for their eventual removal or replacement by other modules. Lastly, the multiple cloning site was redesigned to add a XhoI site and to ensure in-frame reading through all of the added sequences and the transporter-encoding insert.

The method used to isolate recombinant transport proteins from *Xenopus laevis* oocytes is described in the flow diagram of Figure 1B. Briefly, oocytes were extracted from frogs and individualized (day 1), injected with cDNA-derived cRNA encoding for the corresponding recombinant transporter (day 2), incubated at 18°C during days 3 and 4 (when proteins are expressed at their highest levels) and used in various studies thereafter (see below). Isolation of total membranes, membrane solubilization with detergent and affinity chromatography was done on day 5. After overnight protease cleavage at 4°C, transport proteins were eluted (day 6) for further analyses.

### Isolation of egg yolk-depleted total membranes of *X. laevis* oocytes

Egg yolk proteins such as vitellogenins [4] are abundant and sticky molecules hindering drastically detergent solubilization of isolated membranes and protein binding to chromatography resins. Removal of the egg yolk from homogenates was a difficult and challenging step towards the purification of recombinant transport proteins. It was successfully achieved by using the following approach: *i.)* lysis of the oocytes in a salt-free 20 mM Tris-HCl (pH 8; Tris) buffer, *ii.)* differential centrifugation to obtain total membranes and *iii.)* membrane wash in a high-salt (1 M NaCl) Tris buffer. In Figure S1 the effects of these buffers as well as those of other buffers for oocyte lysis and membrane isolation are illustrated. As seen in Figure S1A, lysis of oocytes in high ionic strength Tris buffers, i.e., Tris with 1 M NaCl (NaCl) and Tris with 100 mM MgCl_2_ (MgCl_2_), leads to solubilization of egg yolk proteins, while lysis in salt-free Tris buffers, i.e., Tris with 5 mM EDTA/EGTA (EDTA/EGTA) and Tris only (Tris), does not. The almost complete solubilization of the egg yolk proteins at high ionic strength is clearly seen by the black pellets (color of egg pigments) after low spin centrifugation at 1,000 *g* of the oocyte homogenates (Figure S1A, NaCl and MgCl_2_). In contrast, yellow pellets are found in the EDTA/EGTA and Tris lyses, indicating minor solubilization of the egg yolk proteins. The supernatants collected after the 1,000 *g* centrifugation of the four oocyte lysates (NaCl, MgCl_2_, EDTA/EGTA and Tris) were ultracentrifuged at 150,000 *g* and the total membranes obtained (pellets). SDS-PAGE from the supernatants and pellets after ultracentrifugation is shown in Figure S1B. In agreement with the results displayed in Figure S1A, large amounts of egg yolk proteins, e.g. vitellogenins that migrate at ∼100 kDa [4], were found in supernatants and pellets from high ionic strength lyses (see NaCl and MgCl_2_), and low amounts in those from salt-free lyses (see EDTA/EGTA and Tris). Total membranes from oocyte lysis in Tris buffer contained the smallest amounts of egg yolk contamination (Figure S1B, rightmost lane). Importantly, this contamination was almost completely removed from the preparations through a subsequent wash step of the total membranes in Tris buffer containing 1 M NaCl (Figure S1C, rightmost lane).

### Expression, localization and function of recombinant AQP1 and KCC4 in *X. laevis* oocytes

AQP1 and KCC4 were chosen as model channel and SLC type proteins, respectively, for the study of the structure of purified recombinant transporters expressed in *Xenopus laevis* oocytes. As a first step, we investigated their expression, localization and function in oocytes.

**Figure 2:**
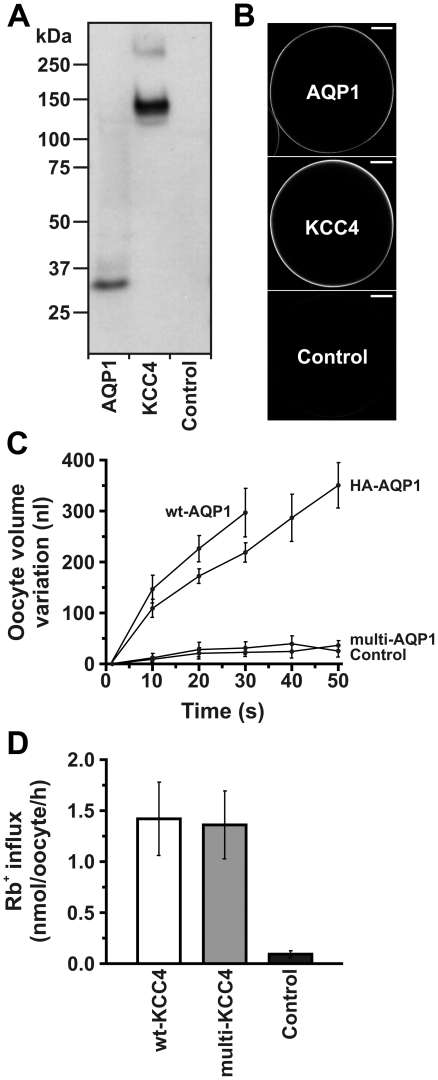
Expression, localization and function of recombinant AQP1 and KCC4 in *X. laevis* oocytes. (A) Cell surface biotinylation experiments and subsequent Western blot analyses using anti-HA antibodies indicated plasma membrane expression of multi-AQP1 and multi-KCC4 (i.e. AQP1 and KCC4 containing a long N-terminal extension as described in Figure 1A): see multi-AQP1 monomer band below the 37 kDa marker, and multi-KCC4 monomer and dimer bands below the 150 kDa and above the 250 kDa markers, respectively. (B) Confocal immunofluorescence microscopy using anti-HA antibodies localized multi-AQP1 and multi-KCC4 in the plasma membrane. These representative images were recorded at 10x magnification. Scale bar: 200 µm. (C) Functional characterization of different AQP1 constructs. Swelling in water of non-injected oocytes (Control) and oocytes injected with cRNA of wt-AQP1 (no N-terminal extension), HA-AQP1 (only N-terminal HA epitope) and multi-AQP1. Multi-AQP1 was not functional while wt-AQP1 and HA-AQP1 had comparable activities. Data correspond to oocyte volume variation measurements in water at different time points. They are shown as averages (± S.E.) of 4-6 experiments. (D) Functional characterization of different KCC4 constructs. Similar levels of Rb^+^ influx into wt-KCC4 and multi-KCC4 expressing oocytes indicated full activity of the recombinant transporter. Data correspond to Rb^+^ influxes and are shown as averages (± S.E.) of 4 experiments (9-15 oocytes/experiment). Oocytes were non-injected (Controls), or injected with 20 ng of AQP1 and KCC4 cRNAs for expression and localization studies (A and B), and injected with 5 ng of AQP1 and 20 ng of KCC4 cRNAs for functional studies (C and D).

Cell surface biotinylation experiments with multi-AQP1 and multi-KCC4 (that is of AQP1 and KCC4 with a long N-terminal extension as described in Figure 1A) expressing oocytes were performed. Subsequent Western blot analyses revealed their expression in the plasma membrane (Figure 2A). Confocal immunofluorescence was consistent with the cell surface localization of those recombinant proteins (Figure 2B).

The functional characterization of AQP1 was carried out by measuring cell volume variations during osmotic swelling in water and that of KCC4 by measuring Rb^+^ influx. Based on these studies, multi-AQP1 was non-functional, the increase in cell volume being similar to that of control oocytes and well below that seen in oocytes expressing untagged and HA-tagged AQP1 (wt-AQP1 and HA-AQP1; Figure 2C). Although these results indicate that the N-terminal extension before the HA epitope abolishes function, it should be noted that the functional HA-AQP1 protein is similar to the truncated multi-AQP1 protein obtained after purification for which the structural organization is not disrupted (as will be shown below). On the other hand, levels of Rb^+^ influx by multi-KCC4 expressing oocytes were similar to those observed for wt-KCC4 expressing oocytes indicating full activity of the recombinant transporter (Figure 2D).

### Purification of recombinant aquaporin-1 and SLC transporters expressed in *X. laevis* oocytes

Egg-yolk depleted total membranes were solubilized with the non-ionic and mild detergent Triton X-100. After removal of the non-solubilized fraction by ultracentrifugation, His-tagged proteins in the supernatant were bound to a cobalt resin and washed with imidazole to diminish background contamination caused by non-specific protein binding. Transporters were then specifically eluted from the cobalt resin by HRV3C protease cleavage directly onto the column instead of by the common elution by high concentrations of imidazole. Because the HRV3C protease is His-tagged, it remains bound to the cobalt resin not contaminating the eluted transport proteins. As expected, purified recombinant proteins had a shorter N-terminus (only HA epitope, see Figure 1A) than the originally expressed forms, e.g. multi-AQP1.

**Figure 3:**
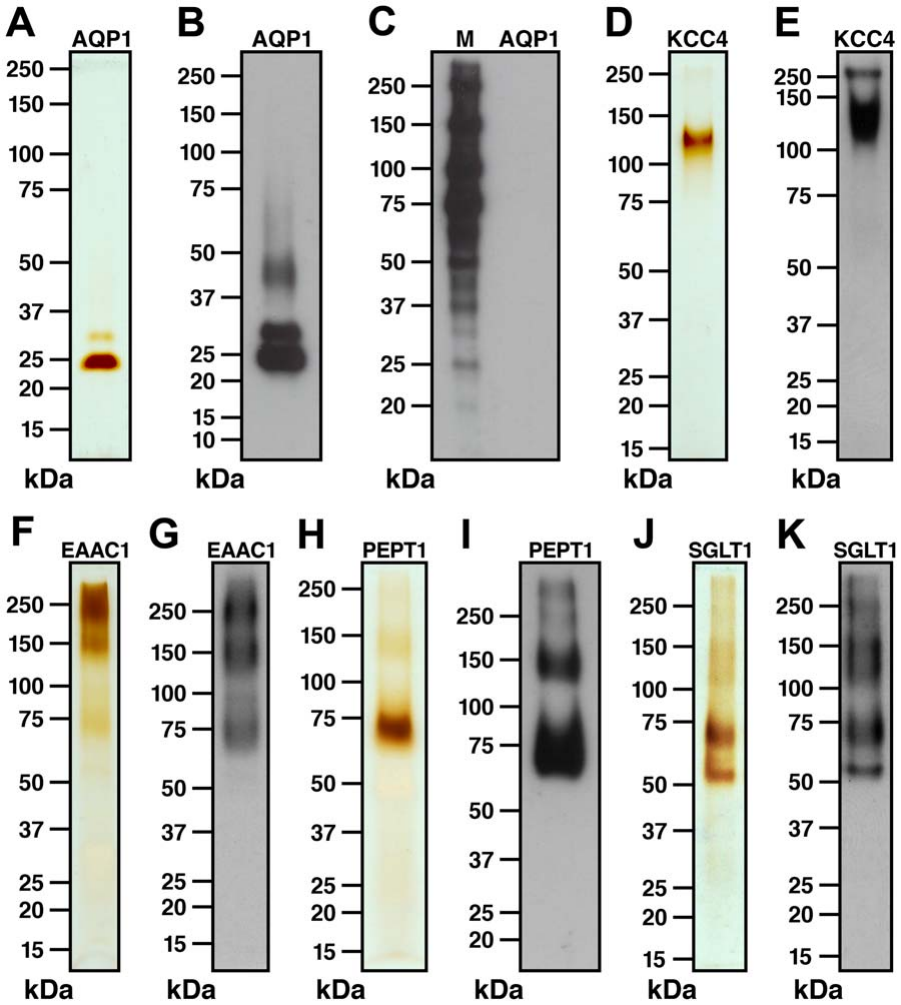
SDS-PAGE, silver-staining and Western blot analyses of purified, recombinant channel and SLC transporters expressed in *X. laevis* oocytes. Silver-stained SDS/polyacrylamide gels (A,D,F,H and J), and Western blots (using anti-HA: B,E,G,I and K and anti-pentahistidine antibodies: C) from representative purifications. (A) and (B) The recombinant, nonglycosylated human AQP1 monomer runs at ∼25 kDa similar to native AQP1 isolated from human erythrocytes [6]. Additional higher molecular mass bands are discerned corresponding to glycosylated and dimeric AQP1 forms. (C) No His-tagged proteins were immunodetected in purified AQP1 preparations by immunoblotting (lane: AQP1). In contrast, strong signals were obtained for the His-tagged protein markers (lane: M; positive control). (D) and (E) Monomer (below 150 kDa marker) and dimer (∼250 kDa) bands of non- and glycosylated, recombinant mouse KCC4 [12], [13]. (F) and (G) Monomeric (∼75 kDa [16]), dimeric (∼150 kDa) and oligomeric glycosylated, recombinant human EAAC1. (H) and (I) Monomeric (∼70 kDa [17]), dimeric (∼140 kDa) and oligomeric non- and glycosylated, recombinant human PEPT1. (J) and (K) Monomeric non- (∼55 kDa [18]) and glycosylated (∼70 kDa), and oligomeric non- and glycosylated, recombinant human SGLT1. All bands on silver-stained SDS/polyacrylamide gels can be assigned to bands observed on corresponding Western blots.

Figure 3 illustrates the high level of purity achieved by this method for AQP1, KCC4, EAAC1, PEPT1 and SGLT1. All of the recombinant transporters (non- and/or glycosylated, and oligomeric forms) were identified on silver-stained SDS/polyacrylamide gels (Figure 3A,D,F,H, and J) and their identity was confirmed by Western blot analyses using anti-HA antibodies (Figure 3B,E,G,I and K). Importantly, no contaminants or protein degradation are found on silver-stained SDS/polyacrylamide gels and Western blots, thus validating the specific purification procedure and indicating correct folding of the proteins. Lastly, immunoblotting using anti-pentahistidine antibodies suggested that the eluted recombinant transporters were free of their His-tags and that His-tagged HRV3C protease did not co-elute (see Figure 3C for AQP1 used as example). In case of persisting protein contaminations, purity could be improved by a second affinity chromatography step based on the FLAG epitope. This purification step would be subsequent to the cobalt affinity chromatography, but eluting the proteins with imidazole instead of by protease cleavage onto the column. As documented in Figure 3, the five tested transport proteins were highly pure, therefore not needing an additional FLAG affinity chromatography step.

Determination of the protein concentration after purification allowed us to estimate the amount of protein expressed per oocyte. Expression levels for our model proteins AQP1 and KCC4 were ∼6 and ∼3 ng per oocyte, respectively. The overall range for the five tested transport proteins was 3–7 ng per oocyte.

### Structural analyses by TEM of purified recombinant AQP1 and KCC4

**Figure 4:**
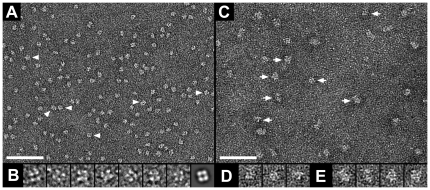
Negative stain TEM of purified recombinant AQP1 and KCC4. The homogeneity of the purified HA-AQP1 protein is reflected in the electron micrograph. Numerous particles exhibit a square shape (arrowheads), which is typical for AQP1 top views [5]. The gallery in (B) displays well-preserved top views of HA-AQP1. The last particle in this gallery (rightmost) is an average calculated from 270 top views. No four-fold symmetry was imposed. In the raw images and average, four densities are clearly visible. The average was low-pass filtered to 15 Å resolution. (C) Overview micrograph of purified HA-KCC4. Two populations of particles are distinct: smaller (minority; arrows to the left) and larger particles (majority; arrows to the right). The former and latter particles were magnified and are displayed in (D) and (E), respectively. The scale bars represent 500 Å (A and C). The frame sizes of the magnified particles are 138 Å (B) and 204 Å (D and E).

Purified HA-AQP1 and HA-KCC4 were adsorbed on parlodion carbon-coated grids, washed, negatively stained and examined by TEM. In Figure 4A the homogeneity of the purified HA-AQP1 can be appreciated. Numerous particles exhibit a square shape (arrowheads) with a side length and diameter of ∼70 Å and ∼100 Å, respectively. In these particles, a central stain-filled pit and four major stain-excluding units are observed: see gallery in Figure 4B for a selection of AQP1 top views. These features are more pronounced in the average calculated from 270 top views of HA-AQP1 (Figure 4B, rightmost frame). The shape, dimensions and structure are in agreement with those of native human AQP1 tetramers purified from erythrocytes [5]. It should be noted that also other slightly different shapes/projections (i.e. not perfectly square) of AQP1 are seen in Figure 4A, because of the several possible particle orientations upon adsorption on a not perfectly flat support film.

On electron micrographs of purified HA-KCC4 (Figure 4C) two populations of particles were found. Smaller particles (marked by arrows to the left) were minor and slightly elliptical with an approximate diameter of ∼100 Å (∼7,850 Å^2^), while larger particles (marked by arrows to the right) were major and rectangular with dimensions of ∼130 Å x ∼100 Å (13,000 Å^2^). Based on the calculated surfaces, the larger particles were twice as big as the smaller particles suggesting two oligomeric forms of KCC4.

### 2D crystallization of AQP1 and analysis of tubular crystals

For 2D crystallization, the detergent Triton X-100 was exchanged by n-decyl-beta-D-maltopyranoside (DM) during protein concentration (see Methods). Because the critical micellar concentration of DM is about 6 times higher than that of Triton X-100, DM is more suitable for fast removal of detergent by dialysis and thus for 2D crystallization.

**Figure 5:**
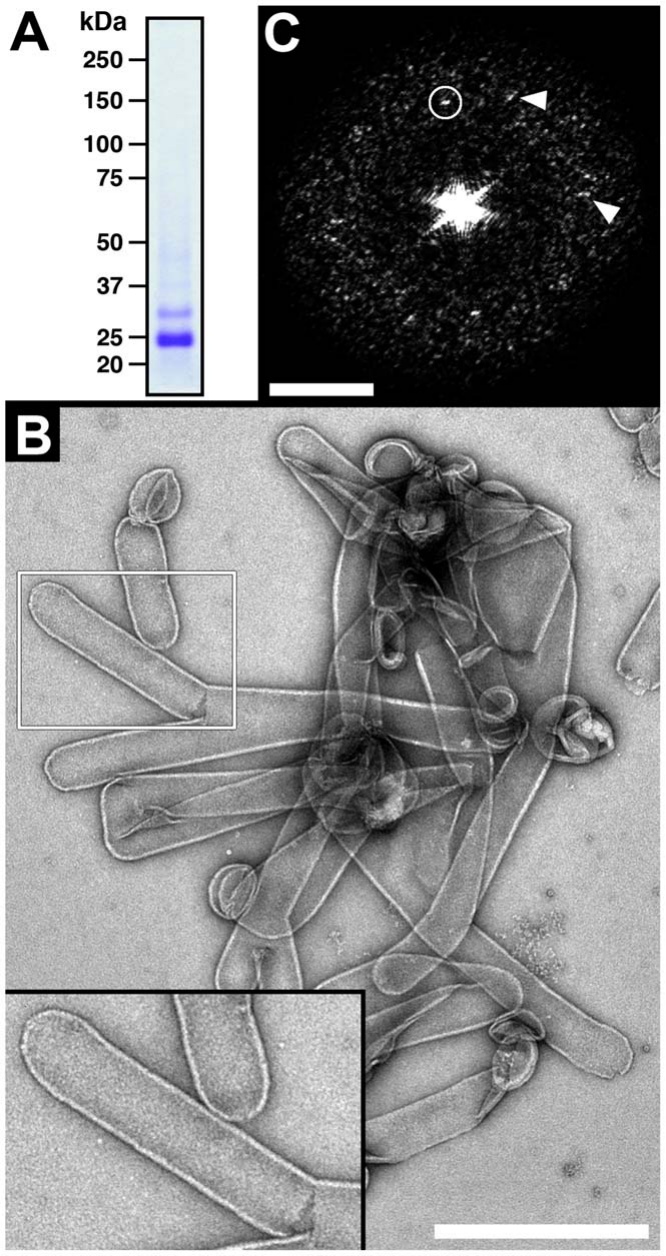
Tubular crystals of recombinant human AQP1. (A) Coomassie Blue-stained SDS/polyacrylamide gel of the purified HA-AQP1 used for 2D crystallization. Nonglycosylated HA-AQP1 migrates at ∼25 kDa while glycosylated proteins migrate above. (B) Negatively stained tubular crystals of HA-AQP1. The area marked by the white box was magnified and is displayed as inset: tubular crystals with a typical width of ∼0.11 µm are seen. (C) The power spectrum calculated from a flattened tubular HA-AQP1 crystal indicates regular order. Strong and weaker diffraction spots are marked by a circle and arrowheads, respectively. The scale bars represent 0.5 µm (B) and (50 Å)^−1^ (C). The frame size of the inset in (B) is 0.503×0.362 µm.

The Coomassie Blue-stained SDS/polyacrylamide gel in Figure 5A shows the high purity of the HA-AQP1 protein used for 2D crystallization. Nonglycosylated HA-AQP1 migrates at ∼25 kDa while glycosylated forms above (compare with Figure 3A and B). Tubular crystals of HA-AQP1 were successfully grown (Figure 5B) using reconstitution conditions similar to those previously reported for 2D crystals of human AQP1 from erythrocytes [6]. Power spectra calculated from flattened HA-AQP1 tubes (Figure 5C) showed strong diffraction spots at ∼ (49 Å)^−1^ (indicated by a circle) and weaker ones at ∼ (44 Å)^−1^ (indicated by arrowheads). Diffraction of our tubular crystals also indicated mosaicity and anisotropy impeding the determination of the crystal lattice parameters and the calculation of a projection structure.

## Discussion

The structure determination of membrane proteins is lagging behind that of water-soluble proteins mainly due to the difficulty in heterologously expressing and isolating the required amounts for structural analyses. Although advances have been achieved over the past years (mainly with prokaryotic membrane proteins), the number of structures of eukaryotic and in particular mammalian polytopic membrane proteins is still negligible. In the various cell types and systems that are currently used to express mammalian membrane proteins, i.e., bacteria, yeast, insect cells, mammalian cell lines and cell-free systems, proteins are often non-functional, mistargeted, misfolded, aggregated or degraded at abnormal rates. Yet, eukaryotic cell expression systems have been shown to be more appropriate than prokaryotic and cell-free systems to generate functional animal proteins because of their specific lipid environment and more elaborated translational and post-translational machineries.

*X. laevis* oocytes are being used very successfully for expression cloning [7], [8] and to study the function of eukaryotic membrane proteins, especially of mammalian transporters [2]. The main advantage of oocytes compared to other cell types is their ability to translate a wide variety of heterologous mRNAs or cDNA-derived cRNAs efficiently and to modify translation products into functional membrane proteins [9]. On the other hand, obtaining significant amounts of purified membrane proteins from this expression system has always been considered a great challenge given that the presence of egg yolk limits or even impedes protein purification.

In this study, we made use of the advantages offered by the *X. laevis* oocyte expression system and established for the first time a method that allows isolating highly pure recombinant mammalian transport proteins. For this novel method, we engineered an expression vector (called pMJB08) provided with all of the necessary features for affinity purification and immunodetection of membrane proteins as well as for the partial removal of the epitope tags introduced (see *Results* and Figure 1A). A challenging but crucial step in the purification procedure was to remove the contaminating egg yolk from membrane preparations. This was achieved by lysing oocytes in a salt-free buffer, separating cell fractions by differential centrifugation and washing total membranes with a high-salt buffer (see *Results* and Figure S1). Through this method, five different transport systems (AQP1, KCC4, EAAC1, PEPT1, and SGLT1) were produced in microgram amounts with no contamination or degradation detectable on silver-stained SDS/polyacylamide gels and by Western blot analyses (Figure 3). The absence of degradation indicated correct folding of the transporters and thus no exposure of unfolded protein domains to proteases. Further studies showed that the two model proteins (AQP1 and KCC4) produced with pMJB08 were expressed and localized at the plasma membrane and that multi-KCC4 was fully functional (Figure 2). Although multi-AQP1 was devoid of functional activity, HA-AQP1 did behave as a wild-type water channel, suggesting that removal of the His-FLAG epitope by the HRV3C protease during purification leads to the formation of an active transport system.

Negative stain TEM and SPA showed highly homogeneous preparations of purified HA-AQP1 in the expected tetrameric form (Figure 4A and B), implying correct protein folding and supramolecular assembly. On the other hand, KCC4 preparations were almost homogeneous, including a major population of larger particles and a minor one of smaller particles (Figure 4C–E). The larger particles were at a size that is consistent with those of homodimers, which are one of the assembly forms taken by all KCC family members as suggested recently by biochemical experiments [10]. The other particles were half as big but exhibited a similar appearance, pointing to a monomer-dimer equilibrium or a partial disruption of the larger particles (dimers) into smaller particles (monomers) during detergent solubilization. It should be noted that this is the first time that a member of the cation-chloride cotransporter (SLC12) family is purified, and that its shape, dimensions, low-resolution structure and oligomeric state is determined by TEM (i.e. by a direct method).

For TEM and SPA, relatively low concentrations of protein are needed, e.g., 2–3 µl per grid of a 30–60 µg/ml protein solution depending on the molecular mass of the protein/complex being analyzed and the desired particle distribution density on the grid. This implies that the method presented here is highly attractive for structural studies of recombinant membrane proteins and their complexes by TEM and SPA. To set up 2D crystallization trials, ∼50 µg of pure HA-AQP1 was prepared from ∼7800 oocytes (Figure 5A). Tubular AQP1 crystals were successfully grown (Figure 5B), and indicated strong and weaker diffraction spots at ∼(49 Å)^−1^ and ∼(44 Å)^−1^, respectively (Figure 5C). These frequencies may be assigned to diffraction spots (±2,0) or (0,±2) at (48 Å)^−1^, and (±2,±1) or (±1, ±2) at ∼(43 Å)^−1^ of a negatively stained human AQP1 2D crystal with typical lattice parameters of a = b = 96 Å, gamma = 90° and p422_1_ symmetry [5]. Importantly, the ability of HA-AQP1 to crystallize is a strong indicator of the structural integrity of the purified recombinant protein.

Currently, we are able to isolate microgram amounts of pure transport protein from manually injected oocytes for TEM, SPA and 2D crystallization. The yield is relatively low compared to other expression systems, but the protein produced is of excellent quality, i.e. homogeneous, correctly folded/assembled, and neither prone to degradation nor aggregation. In the future, automated injection of several thousands oocytes per hour by commercial robots, now up to 600 by Roboinject (Multi channel Systems, Germany) [11], will allow purification of enough mammalian recombinant transport proteins to grow 3D crystals for structure determination by X-ray crystallography. To us, this prediction is realistic considering that it is now possible to carry out 3D crystallization trials with 250–750 ng of protein per conditions, e.g. in 50 nl drops.

## Materials and Methods

All animal experiments were in accordance with the Swiss Animal Welfare Law and were approved by the Local Veterinary Authority Bern (Veterinäramt Bern; Permit Number: 110/08).

### Expression vector

For the heterologous expression of transporters in *X. laevis* oocytes and their subsequent affinity purification and immunodetection, we modified the Pol1 expression vector [3] by adding an N-terminal extension as described in *Results* and Figure 1A. This new vector named pMJB08 was generated by the insertion of two prehybridized complementary oligonucleotide fragments, which possess single-stranded cohesive, and XmaI-NcoI- and NcoI-HindIII-compatible ends, into a XmaI-HindIII-treated Pol1 vector fragment. The two upper oligonucleotides and their complementary oligonucleotides are found in Table S1. Fragments were ligated with the Rapid DNA Ligation Kit (Roche) and the vector was propagated in XL1-Blue Cells (Stratagene).

### cDNA constructs

cDNAs from KCC4 (mouse), and AQP1, EAAC1, PEPT1 and SGLT1 (human) were cloned by PCR (using *Pfu Turbo* DNA Polymerase, Stratagene) from carrier cDNA constructs of our laboratory. The oligonucleotides used for PCR reactions are found in Table S1. All digested cDNAs were introduced in-frame into the pMJB08 expression vector.

### Protein expression in *X. laevis* oocytes

Ovarian tissue was surgically removed from the frogs and treated with 3 mg/ml of collagenase A (Roche) for 2 h in Modified Barth's Medium (MBM: 88 mM NaCl, 1 mM KCl, 2.4 mM NaHCO_3_, 0.82 mM MgSO_4_, 0.66 mM NaNO_3_, 0.75 mM CaCl_2_, 10 mM HEPES-NaOH (pH 7.4)) but without CaCl_2_ and supplemented with P/S antibiotics (GIBCO™ Penicillin-Steptomycin liquid, Invitrogen). This buffer is isoosmolar relative to the *X. laevis* plasma (∼200 mOsm). Subsequently, defolliculated stage V-VI oocytes were isolated, transferred into plates containing MBM medium supplemented with P/S antibiotics and maintained at 18°C prior to injection. Depending on the experiment, oocytes were injected with 5 or 20 ng of cDNA-derived cRNA (*in-vitro* transcription with the mMESSAGE mMACHINE® T7 kit, Ambion) and maintained for 3–4 days at 18°C in MBM supplemented with P/S antibiotics.

### Isolation of egg yolk-depleted total membranes from oocytes

Between 350–400 oocytes were homogenized in 9 ml of 20 mM Tris-HCl (pH 8) supplemented with the Complete® protease inhibitor cocktail (Roche). To remove cell debris, nuclei and pigments, the homogenate was subjected to a low spin centrifugation (1,000 *g*; 15 min; 4 °C). The supernatant was harvested with a glass Pasteur pipette, transferred into an ultracentrifuge tube and subjected to ultracentrifugation at 150,000 *g* (1 h; 4 °C). The pellet containing total membranes was homogenized in 9 ml of high ionic strength buffer (20 mM Tris-HCl (pH 8), 1 M NaCl) supplemented with Complete® and ultracentrifuged at 150,000 *g* (1 h; 4 °C).

### Protein purification

The pellet of egg yolk-depleted total membranes from the precedent ultracentrifugation was homogenized/solubilized in 9 ml of solubilization buffer: 20 mM Tris-HCl (pH 8), 150 mM NaCl, 0.01% NaN_3_ and 2% Triton X-100 supplemented with Complete® and incubated on a rotator (1 h; 4°C). The non-solubilized fraction was removed by ultracentrifugation at 150,000 *g* (1 h; 4 °C). The supernatant containing the detergent-solubilized His-tagged transport protein was incubated with pre-equilibrated HisPur™ cobalt resin (250 µl bed volume; Thermo Scientific) on a rotator (3 h; 4 °C; binding step) and subsequently transferred into a spin column (Promega). His-tagged proteins bound to cobalt resin were washed thoroughly in three steps: *i.)* 20 ml of washing buffer: 20 mM Tris-HCl (pH 8), 150 mM NaCl, 0.01% NaN_3_, 0.05% Triton X-100 and 10 mM imidazole; *ii.)* 20 ml of washing buffer with 20 mM instead of 10 mM imidazole; and *iii.)* 10 ml of cleavage buffer: 20 mM Tris-HCl (pH 8), 150 mM NaCl, 0.025% Triton X-100. The column was allowed to drip and empty without letting the resin run dry. The tip of the spin column was then cut and 300 µl of cleavage buffer containing 20 units of HRV3C (BioVision) were added. After sealing with Parafilm M, the tip was mounted on a rotator and agitated to favor proteolytic cleavage (overnight; 4 °C). Truncated, HA-tagged proteins were eluted by a low spin centrifugation at 960 *g* (3 min; 4°C). Four to six purifications (i.e. one corresponds to protein purified from 350–400 oocytes) were pooled in an Amicon® Ultra 100 K concentrator (Millipore) and concentrated to a final volume of ∼100 µl. The protein concentration was determined using the BCA™ protein assay kit (Pierce, Perbio Science).

### SDS-PAGE and Western blot analyses

Purified proteins were boiled in 5x sample buffer (without reducing agent) and run on 6% Tris-Tricine SDS/polyacrylamide gels. Gels were either stained (silver or Coomassie Blue) or transferred onto Immobilon-P membrane blots (Millipore) for Western blot analysis. Blots were sequentially incubated with primary and secondary antibodies. The primary antibodies, mouse monoclonal anti-HA (Sigma) and anti-Penta-Histidine (Qiagen), were used at a dilution of 1/1,000. The secondary antibody, HRP-conjugated goat anti-mouse anti-IgG (Bio-Rad), was used at a dilution of 1/4,000.

### Cell surface biotinylation

For each experiment, twenty intact oocytes were incubated (1 h; 4°C) with 1.5 mg/ml sulfo-LC-NHS-(+)-Biotin (Pierce), washed with quenching solution (100 mM glycine) and lysed in 1 ml of 50 mM Tris-HCl (pH 7.4), 150 mM NaCl, 1% Triton X-100, 0.5% Na-deoxycholate, 0.1% SDS supplemented with Complete®. After incubation (2 h; 4°C) and centrifugation at 18,000 *g* (15 min; 4 °C), supernatants were transferred into new tubes. Cell surface biotinylated-proteins were purified by addition of 50 µl of streptavidin agarose beads (Pierce), overnight incubation at 4°C, and recovery of the beads by centrifugation at 9,000 *g* (1 min; 4 °C). Finally, proteins were released from the beads by boiling in 2x sample buffer, and subjected to SDS-PAGE and Western analyses as described previously.

### Immunofluorescence studies

Oocytes were fixed with 4% paraformaldehyde for 20 min and permeabilized with 0.3% Triton X-100 for 5 min at room temperature. After 1 h incubation in blocking solution (PBS with 5% goat serum, 0.5% bovine serum albumin, and 0.02% NaN_3_), oocytes were incubated for another hour with rabbit polyclonal anti-HA antibodies (dilution 1/200; Sigma) and for an additional hour with Alexa Fluor® 594-conjugated goat anti-rabbit anti-IgG antibodies (dilution 1/4,000; Invitrogen). Images were recorded with a C1 confocal laser microscope system (Nikon).

### Cell volume measurements

Oocytes expressing wt-AQP1, HA-AQP1 and multi-AQP1 were incubated in water and volume variations were measured during osmotic swelling with a SZ61 stereomicroscope (Olympus) equipped with a DP20 digital camera (Olympus). Cell volumes were calculated from cell radii measured at different time points. Volumes in nanoliters (nl) were calculated from radii (r) measured in mm, with the formula (4/3 × π × (r/10)^3^) × 10^6^.

### Influx studies

The function of wt-KCC4 and multi-KCC4 expressing oocytes was determined as previously described [12], [13]. Briefly, oocytes were incubated for 1 h in a low Cl^−^/hypotonic solution (∼5 mM Cl^−^ and 125 mOsm) to activate KCC4, incubated for another 45 min in a physiological solution (86 mM Cl^−^ and ∼200 mOsm) supplemented with 5 mM Rb^+^ (used as a tracer of K^+^) and 10 mM ouabain, and bathed several times in a wash solution supplemented with 10 mM ouabain, 250 mM bumetanide and 250 mM furosemide. Cells were then lysed in pure nitric acid and their Rb^+^ content was measured by atomic absorption spectrometry. The data obtained were converted to fluxes (nmol/oocyte/h). All steps were carried out at room temperature.

### 2D crystallization of AQP1

Eluates from twenty-one purifications (∼7,800 oocytes, about 4 weeks of work) were pooled and concentrated to a final volume of ∼100 µl. The detergent Triton X-100 was exchanged for DM in the concentrator by addition of 400 µl of 20 mM Tris-HCl (pH 8), 150 mM NaCl, 0.15% DM to the ∼100 µl of concentrated protein, and concentrating back to a volume of ∼100 µl. This was repeated ten times to ensure complete exchange. The final volume of ∼100 µl had a protein concentration of ∼0.5 mg/ml. For 2D crystallization, purified HA-AQP1, i.e. truncated multi-AQP1, was mixed with *Escherichia coli* polar lipids solubilized in 1% DM, 20 mM Tris-HCl (pH 8), 150 mM NaCl, 0.01% NaN_3_ at lipid-to-protein ratio of 0.5, 0.75 and 1. 2D crystallization of HA-AQP1 was performed by dialysis of the protein/lipid/detergent mixture at 24 °C for 14 days. The detergent-free dialysis buffer was 20 mM Mes-NaOH (pH 6), 200 mM NaCl, 10 mM MgCl_2_, 2 mM DTT, 10% glycerol, 0.01% NaN_3_ as previously described for the 2D crystallization of human AQP1 isolated from red blood cells [6].

### Transmission electron microscopy

Detergent-solubilized transport proteins were adsorbed for ∼10 s to parlodion carbon-coated copper grids rendered hydrophilic by glow discharge at low pressure in air. Grids were washed with three drops of double distilled water and stained with 2 drops of 0.75% uranyl formate. Grids of 2D AQP1 crystals were prepared similarly but included a longer adsorption time of ∼60 s. Electron micrographs were recorded with a Philips CM12 TEM operated at 80 kV and equipped with a Morada 11 megapixel CCD camera.

### SPA and image processing

SPA of purified HA-AQP1 and the calculation of the top view in Figure 4B (rightmost panel) was performed using the EMAN software package [14]. The power spectrum in Figure 5C of a flattened tubular HA-AQP1 crystal was calculated using the SEMPER system [15].

## Supporting Information

Figure S1 **Isolation of egg yolk-depleted total membranes of** ***X. laevis*** **oocytes.** (A) Homogenization of oocytes in four different lysis buffers: *i.)* 20 mM Tris-HCl (pH 8; Tris) with 1 M NaCl (NaCl); *ii.)* Tris with 100 mM MgCl_2_ (MgCl_2_); *iii.)* Tris with 5 mM EDTA/EGTA (EDTA/EGTA) and *iv.)* Tris only. The pellets after low spin centrifugation at 1,000 *g* of the homogenates are displayed. Buffers with high ionic strengths (NaCl and MgCl_2_) solubilized egg yolk proteins, resulting in black pellets mainly containing egg pigments. Salt-free buffers (EDTA/EGTA and Tris) yielded yellow pellets characteristic of the egg yolk. The four supernatants were collected and ultracentrifuged at 150,000 *g* (150 k*g*). (B) SDS-PAGE of the supernatants (S) and pellets (P) after ultracentrifugation. Consistent with the results in (A), supernatants and pellets from lyses at high ionic strength contained large amounts of egg yolk contaminants, e.g. vitellogenins migrating at ∼100 kDa. In contrast, vitellogenins were absent and weakly present in supernatants and pellets from homogenates prepared in salt-free buffers. The best result was obtained with Tris buffer: see rightmost lane. (C) The weak vitellogenin contamination in the pellet (see lane between blue lines in (C) and lane P 150 k*g* Tris in (B)) was almost completely removed by the addition of a NaCl wash step. The beneficial effect of this wash step for the purity of total membranes can directly be compared with that of a wash with Tris buffer (see lanes labeled ‘Wash’ in the panel). The rightmost lane illustrates the isolation of total membranes under the best conditions. The lane between the two blue lines is from the same gel but from a different well. All gels were stained with Coomassie Blue.
(TIF)

Table S1 **Synthetic oligonucleotides used to generate the pMJB08 vector and for PCR reactions.** All oligonucleotides are written 5′ to 3′ excepted for the two complementary oligonucleotides that are written 3′ to 5′. Bold letters designate cohesive compatible enzyme restriction sites or enzyme restriction sites in PCR oligonucleotides. S = sense, AS = anti-sense.
(DOC)
