# Supplementary figures and images for: Frog Oocytes to Unveil the Structure and Supramolecular Organization of Human Transport Proteins
Source: PLoS One. 2011 Jul 7;6(7):e21901. doi: 10.1371/journal.pone.0021901 (PMC3131388; doi:10.1371/journal.pone.0021901)

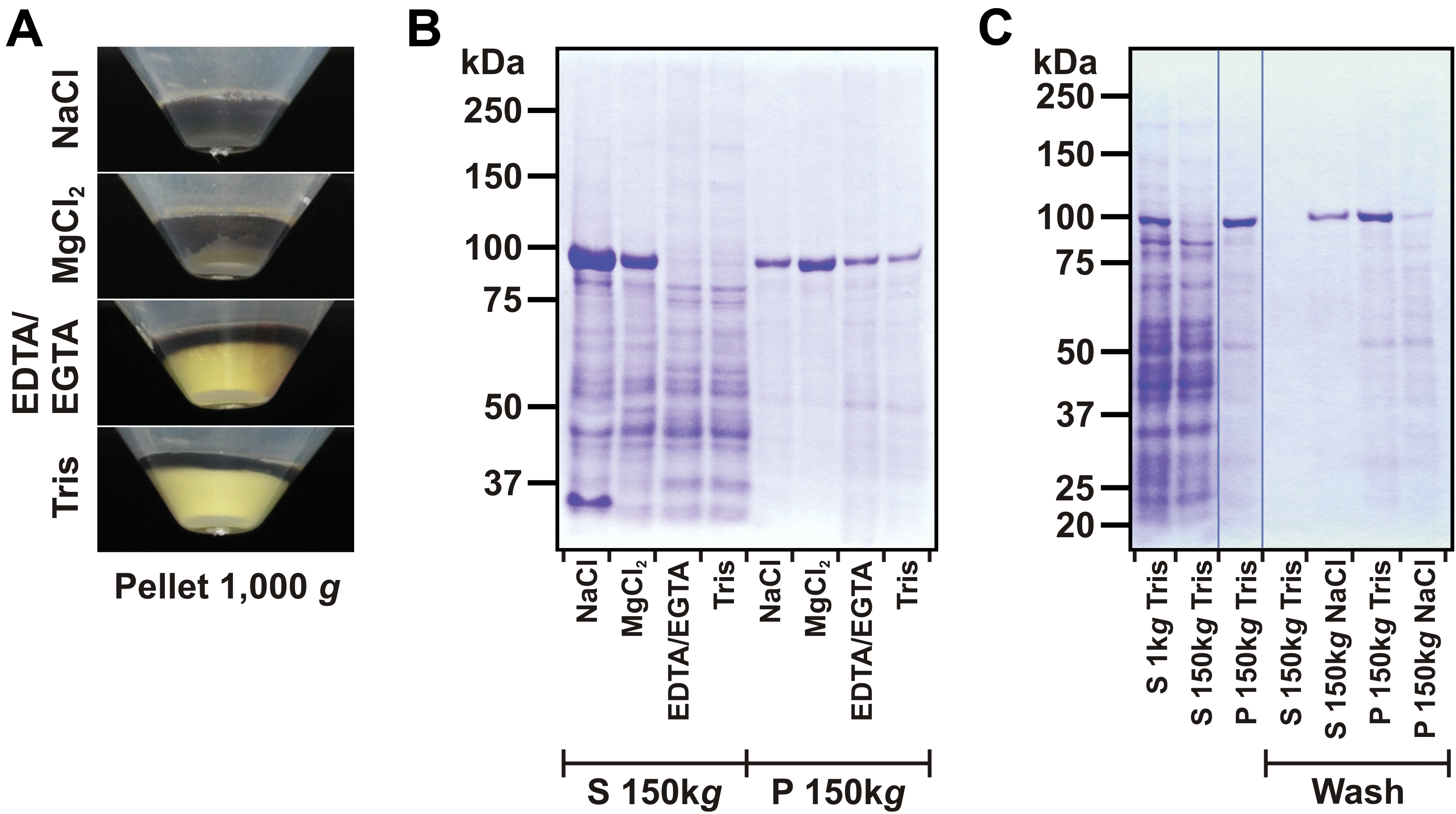

Supplement: Figure S1 — Isolation of egg yolk-depleted total membranes of X. laevis oocytes. (A) Homogenization of oocytes in four different lysis buffers: i.) 20 mM Tris-HCl (pH 8; Tris) with 1 M NaCl (NaCl); ii.) Tris with 100 mM MgCl2 (MgCl2); iii.) Tris with 5 mM EDTA/EGTA (EDTA/EGTA) and iv.) Tris only. The pellets after low spin centrifugation at 1,000 g of the homogenates are displayed. Buffers with high ionic strengths (NaCl and MgCl2) solubilized egg yolk proteins, resulting in black pellets mainly containing egg pigments. Salt-free buffers (EDTA/EGTA and Tris) yielded yellow pellets characteristic of the egg yolk. The four supernatants were collected and ultracentrifuged at 150,000 g (150 kg). (B) SDS-PAGE of the supernatants (S) and pellets (P) after ultracentrifugation. Consistent with the results in (A), supernatants and pellets from lyses at high ionic strength contained large amounts of egg yolk contaminants, e.g. vitellogenins migrating at ∼100 kDa. In contrast, vitellogenins were absent and weakly present in supernatants and pellets from homogenates prepared in salt-free buffers. The best result was obtained with Tris buffer: see rightmost lane. (C) The weak vitellogenin contamination in the pellet (see lane between blue lines in (C) and lane P 150 kg Tris in (B)) was almost completely removed by the addition of a NaCl wash step. The beneficial effect of this wash step for the purity of total membranes can directly be compared with that of a wash with Tris buffer (see lanes labeled ‘Wash’ in the panel). The rightmost lane illustrates the isolation of total membranes under the best conditions. The lane between the two blue lines is from the same gel but from a different well. All gels were stained with Coomassie Blue. (TIF) [file pone.0021901.s001.tif]
