# Supplementary material for: Frog Oocytes to Unveil the Structure and Supramolecular Organization of Human Transport Proteins
Source: PLoS One. 2011 Jul 7;6(7):e21901. doi: 10.1371/journal.pone.0021901 (PMC3131388; doi:10.1371/journal.pone.0021901)
Supplement: Table S1 — Synthetic oligonucleotides used to generate the pMJB08 vector and for PCR reactions. All oligonucleotides are written 5′ to 3′ excepted for the two complementary oligonucleotides that are written 3′ to 5′. Bold letters designate cohesive compatible enzyme restriction sites or enzyme restriction sites in PCR oligonucleotides. S = sense, AS = anti-sense. (DOC) [file pone.0021901.s002.doc]

**Oligonucleotide sequences**

Upper oligo of the first pMJB08 prehybridized fragment:

5’(**XmaI**)**CCGGG**ATGCATcaccaccatcatcaccatcaccaccacGTCGACgattacaaggatgacgacgataagGCGGCCGCGctggaagttctgttccaggggccc**C**(**NcoI**)3’

Complementary oligo of the first pMJB08 prehybridized fragment:

3’(**XmaI**)**C**tacgtagtggtggtagtagtggtagtggtggtgCAGCTGctaatgttcctactgctgctattcCGCCGGCGCgaccttcaagacaaggtccccggg**GGTAC**(**NcoI**) 5’

Upper oligo of the second pMJB08 prehybridized fragment:

5’(**NcoI**)**CATGG**tacccatacgacgtcccagactacgctCCCGGGggatccCTCGAGgaattcTCTAGA**a**(**HindIII**) 3’

Complementary oligo of the second pMJB08 prehybridized fragment:

3’(**NcoI**)**C**atgggtatgctgcagggtctgatgcgagggccccctagggagctccttaagagatct**ttcga**(**HindIII**) 5’

Oligos to produce in-frame carrier cDNA:

hAQP1: hEAAC1:

S (**BamHI**) ccc**ggatcc**ATGGCCAGCGAGTTCAAGAAG S (**BamHI**) cgg**ggatcc**ATGGGGAAACCGGCGAGGAAAGG

AS (**EcoRI**) ccc**gaattc**CTATTTGGGCTTCATCTCCAC AS (**EcoRI**) ccc**gaattc**CTAGAACTGTGAGGTCTGGGTG

mKCC4: hSGLT1:

S (**EcoRI**) gc**gaattc**ATGCCCACGAACTTTACGGTG S (**XhoI**) ggg**ctcgag**ATGGACAGTAGCACCTGGAGC

AS (**HindIII**) ggg**aagctt**GAGGCGCCAGAGTGCAGTGG AS (**HindIII**)ggg**aagctt**TCAGGCAAAATATGCATGGC

hPEPT1:

S (**BamHI**)ggg**ggatcc**ATGGGAATGTCCAAATCACAC

AS (**XhoI**) ggg**ctcgag**TCACATCTGTTTCTGTGAATTGGCC
